# Identifying essential proteins in dynamic protein networks based on an improved *h*-index algorithm

**DOI:** 10.1186/s12911-020-01141-x

**Published:** 2020-06-17

**Authors:** Caiyan Dai, Ju He, Kongfa Hu, Youwei Ding

**Affiliations:** College of Artificial Intelligence and Information Technology, Nanjing University of Chinese Medicine University, Nanjing, 210000 China

## Abstract

**Background:**

The essential proteins in protein networks play an important role in complex cellular functions and in protein evolution. Therefore, the identification of essential proteins in a network can help to explain the structure, function, and dynamics of basic cellular networks. The existing dynamic protein networks regard the protein components as the same at all time points; however, the role of proteins can vary over time.

**Methods:**

To improve the accuracy of identifying essential proteins, an improved *h*-index algorithm based on the attenuation coefficient method is proposed in this paper. This method incorporates previously neglected node information to improve the accuracy of the essential protein search. Based on choosing the appropriate attenuation coefficient, the values, such as monotonicity, SN, SP, PPV and NPV of different essential protein search algorithms are tested.

**Results:**

The experimental results show that, the algorithm proposed in this paper can ensure the accuracy of the found proteins while identifying more essential proteins.

**Conclusions:**

The described experiments show that this method is more effective than other similar methods in identifying essential proteins in dynamic protein networks. This study can better explain the mechanism of life activities and provide theoretical basis for the research and development of targeted drugs.

## Background

With the increasing amount of available medical information, the identification of key proteins has become an area of interest for many researchers [1–3].

In recent years, methods using different perspectives have been developed to mine essential nodes in complex networks. Wang et al [4]. proposed an effective method to identify vertices in dynamic networks using local detection and update strategies. This method locally detects change vertices in a dynamic network and locally updates the influence measure of these change vertices, without globally calculating the influence of all vertices. Essential proteins are those that play an important role in protein evolution and are similar to the definition of essential GO terms presented by Wan et al. [5] Li et al. [6] proposed a new method for identifying essential proteins by combining information on protein complexes and protein–protein interaction (PPI) network topological features. By analyzing the relationship between protein complexes and essential proteins, it was found that proteins in multiple complexes were more likely to be essential than those in only one complex. Based on a statistical analysis of proteins and protein complexes, Luo et al. [7] proposed a method for predicting essential proteins in PPI networks based on the local interaction density and protein complexes. Hu et al. [8] proposed a new method, the E-Burt method, which can be applied to weighted networks. This method fully considers the total connection strength, the number of connection edges, and the distribution of the total connection strength on the connection edge in the local range. Wang et al. [9] used the iterative information of k-shell decomposition to distinguish the influence ability of nodes with the same k-shell. Lei et al. [10] proposed an essential protein exploration method named RWEP using a random walk algorithm that integrates topological and biological properties to determine protein essentiality in PPI networks. Many of the key factors to measure nodes in complex networks are based on graph theory to quantify the topological structure and attributes of each node, and comparisons of the centrality of each node are made through different centrality calculation methods, such as the degree center, median center, proximity center, and edge clustering coefficient center. Quantitative methods can also be used to find the essential nodes in networks [11–14]

The identification of essential proteins in dynamic protein networks reveals those that play the most important role in the evolution of proteins. In the search for essential proteins, the above methods consider only the importance of the nodes themselves to illustrate their centrality, ignoring structural information of network graphs. When modeling the essential proteins, some algorithms treat the protein situation at different time points as the same. However, in the process of protein evolution, the role of proteins can vary over time. Therefore, adding the attenuation coefficient can help to find proteins that are essential in the protein evolution process. The importance of the node itself and other structure information of the network can be combined by the attenuation coefficient to examine the importance of a particular node as it relates to the whole network.

## Methods

Essential proteins in a protein network are usually located at the center of the entire network. The appearance or disappearance of these proteins has a crucial impact on the whole protein network [15–17] Accurately identifying essential proteins in a dynamic protein network is helpful for understanding various biological processes from a systematic point of view, and this information can be widely used to explore the pathogenesis of diseases and to predict and evaluate corresponding treatments. This information can also be used to find new drug targets and open new avenues for drug research and development. Although effective methods have been applied to identify essential proteins in protein interaction networks based on data mining, machine learning, and artificial neural networks, it is still necessary to carry out in-depth research on algorithms to improve the accurate identification of such proteins.

1. Time series on dynamic protein networks

When modeling dynamic protein networks, gene expression data and large-scale static protein networks are usually considered together. The gene expression arrays of *M* genes at *T* time points can be divided into *T* sets. Each set represents the state of *M* genes at the same time point and can be combined into a dynamic protein network based on a time series.

(2) Evolution of proteins

**Fig. 1:**
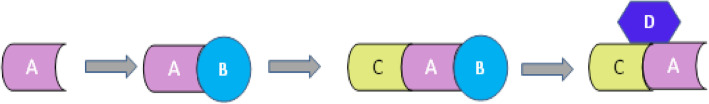
Simple protein evolution

Different protein interaction networks are present at different time points. Figure 1 shows a simple protein evolution process, where A, B, and C represent different proteins that appear at different times in protein evolution.

The ultimate goal of the model is to facilitate subsequent research by identifying essential proteins that play a crucial role in protein evolution or by predicting the linkages between proteins in subsequent points in evolution. This requires recording the evolution of the protein itself. In the past, link relationships between proteins in a network at different time points were recorded as 1 and relationships without links were recorded as 0. This approach is not amenable to a time series, because over time the historic protein data becomes less prominent and recently generated links between proteins play a larger role.

Dynamic protein networks represent the implementation of the entire evolutionary process over time. If a link relationship between proteins is considered to be constant in the evolutionary process over time, it will obviously affect subsequent studies that are based on dynamic protein models. Therefore, it is worth exploring how to incorporate protein link relationships that change over time into the scope of a model so as to correctly identify key proteins in dynamic protein networks and predict future protein link relationships.

(3) The *h*-index method

The *h*-index [18] is a new method for evaluating academic achievements. The *h* stands for “high citation times”. The *h-*index means that a scientist has at most *h* papers cited at least *h* times. The *h*-index was originally used to accurately reflect a person’s academic achievements. A higher *h-*index indicates a greater academic influence. In the study of dynamic protein networks, the *h*-index can be used to find essential proteins, and formula (1) can be used to calculate the *h*-index value of nodes.

1 $$ H- index\left({v}_i\right)=H\left({d}_{u1},{d}_{u2},\dots, {d}_{ud_i}\right)\kern2em uj\in neighbor\left({v}_i\right) $$

where *d*_*i*_ denotes the degree of node *v*_i_, formula *h* (*x*_1_, *x*_2_,... *x*_n_) returns the maximum value of *y*, and at least *y* items from *x*_1_, *x*_2_,... *x*_n_ are greater than or equal to *y*.

However, the *h*-index considers only the importance of the node itself to illustrate its centrality, ignoring some network structure information, which will reduce the accuracy of the node expansion.

**Fig. 2:**
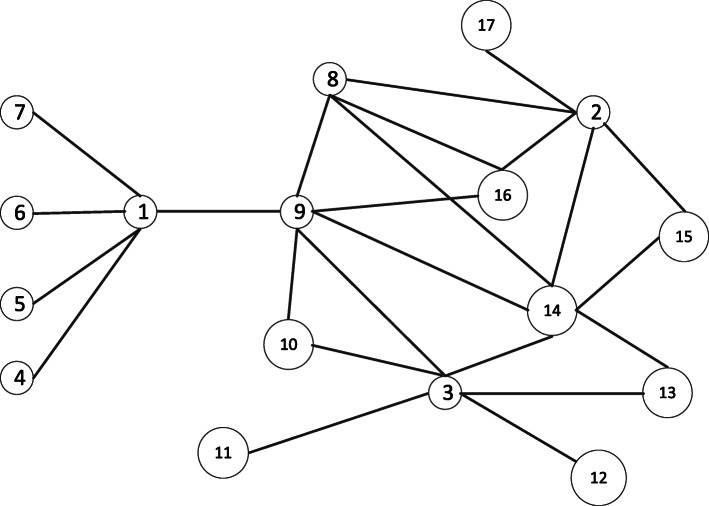
An example of node centrality. **a**. Integrating gene expression data and extracting dynamic proteins. **b**. Combining an open protein network and dynamic proteins to form a dynamic protein network. **c**. Constructing dynamic protein networks at different time points. **d**. The weight of the same edge at adjacent times

For example, in Fig. 2, the centrality of node 2 is relatively large. However, considering the information of nodes in this graph, the expansion ability of node 3 is greater than that of node 2. The reason for this is that when defining the centrality of the *h*-index, some information about the node neighbors is ignored. For example, nodes with a degree less than *y* are completely ignored, resulting in reduced specification accuracy when the node is expanded. Therefore, this method is not accurate in calculating the expansion capacity of nodes.

Therefore, it is necessary to improve the existing *h*-index algorithm on the basis of the established dynamic protein network model to accurately identify key proteins in the network by combining information of the nodes themselves and structural information that has been neglected in previous algorithms.

(4) Monotonicity

The ability to distinguish nodes with different scalabilities and nodes with uniform distribution at different levels is one criterion for evaluating the ranking methods of influential nodes in social networks [8] Monotonicity is used to test the recognition ability of this method for nodes with different extensibility. Formula (2) is used to calculate the m value of ranking Table R. In this equation, n is the number of column groups in list *R* and the number of nodes in column group R. The value of M is always in the range 0–1. Large numbers indicate that nodes have high recognition ability.

2 $$ M(R)={\left(1-\frac{\sum \limits_{r\in R}{n}_r\ast \left({n}_r-1\right)}{n\ast \left(n-1\right)}\right)}^2 $$

After establishing a dynamic protein network model based on attenuation coefficients, the essential protein recognition methods can be investigated. The methods to be adopted are as follows.

1. Construct the whole protein evolution process network based on the attenuation coefficient.

In this network, the weights of each side at the corresponding time points should be added together to obtain the final weights; the formulas are stated below.

In the process of construction, the same edge appears at different times. At the current time point, the corresponding weight calculation will vary. The earlier the edge appears, the more its role in the protein evolution process will change over time. The calculation method of weight corresponding to the edges at each time is as follows:

For each edge (*u*, *v*) in the protein network at time *t*, its weight *D* (*u*, *v*, *t*) varies with time *t* and is defined as:

3 $$ D\left(\left(u,v\right),t\right)=\left\{\begin{array}{c}\delta \left(\left(u,v\right),0\right)\ t=0\\ {}D\left(\left(u,v\right),t-1\right)\ast \lambda +\delta \left(I,t\right)\  otherwise\end{array}\right. $$

in which

4 $$ \delta \left(\left(u,v\right),t\right)=\left\{\begin{array}{c}1\ a(t)\  include\ edge\ \left(u,v\right)\\ {}0\  otherwise\end{array}\right. $$

where *a* (*t*) is a set of all the edges appearing at time *t*; *a* (*t*) is a constant and is called the attenuation coefficient.

The weight of each vertex neighbor is calculated by the weight of the edge. If the vertex *v* is a neighbor of *u*, the weight *w* (*u*, *v*) of *v* for *u* is defined as:

5 $$ w\left(u,v\right)=\frac{D\left(u,v,t\right)}{\sum \limits_{x\in N(u)}D\left(u,\mathrm{x},t\right)} $$

It can be seen from the above definition that $\sum \limits_{v\in N(u)}\mathrm{w}\left(u,v,t\right)=1$ and *w* (*u*, *v*) and *w* (*v*, *u*) are not necessarily equal.

In this way, the weight of each edge in the protein network at different times is will vary.

**Fig. 3:**
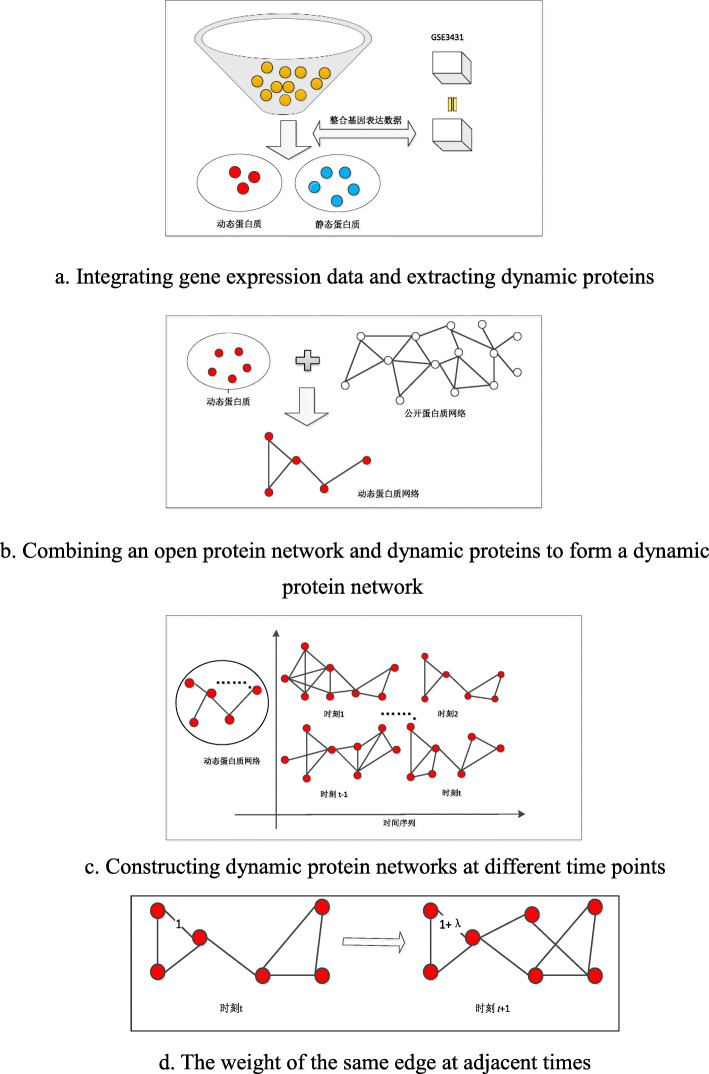
Construction of a dynamic protein network based on the attenuation coefficient

The process of constructing a dynamic protein network based on attenuation coefficients is shown in Fig. 3.

Considering that proteins will change with time in the process of protein evolution, the protein network model is more objective and conforms to the process of biological evolution.

(2) Calculating the cumulative centrality of the node neighborhood

Although the *h*-index measure attempts to determine the centrality of nodes based on the importance of adjacent nodes, some information about the adjacent nodes is still ignored. The centrality of a node can be standardized by using all the information of its adjacent nodes. For this purpose, the cumulative function in definition 1 is used.

### Definition 1.

The cumulative function *c*_*k*_ (*v*_*i*_) is defined as the number of nodes whose *v*_*i*_ neighbors are moderately larger than or equal to k, expressed as follows:

6 $$ {c}_k\left({v}_i\right)=\mid \left\{{v}_j|{v}_j\in {N}_i\kern0.5em and\kern0.5em {d}_j\ge k\right\}\mid $$

The *h*-index function is improved to the cumulative function defined in eq. (7):

7 $$ h- index\left({v}_i\right)={}_k{}^{argmax}\left({c}_k\left({v}_i\right)\ge k\right) $$

This function takes the maximum value of *k*, where the degree of *k* neighbors is greater than or equal to *k* when other cumulative values are ignored by the *h*-index function. Therefore, in the proposed metric, the cumulative value of neighboring nodes is determined for all different values of *k* and is used to standardize node centrality.

In the traditional *h*-index, the weight of each neighbor’s link is considered as 1. In our case, the weight of each neighbor’s link *w*(*u,v*) is different. Therefore, when extending the traditional *h*-index, the extended cumulative function *c*_*k*_ (*v*_*i*_) is defined as follows:

### Definition 1.

Extended cumulative function *c*_*k*_ (*v*_*i*_):

8 $$ {c}_k\left({v}_i\right)=\sum \limits_{v_j\in {N}_k\left({v}_i\right)}\mathrm{w}\left({v}_i,{v}_j\right) $$

Here, *N*_*k*_ (*v*_*i*_) is the set of neighbor vertices whose degree is *k*.

Definition 2, the cumulative function vector *S* (*v*_*i*_), contains the cumulative values of nodes adjacent to node *v*_*i*_ at different degrees. The calculation is shown in formula (9):

9 $$ s\left({v}_i\right)=\left\{{c}_1\left({v}_i\right),{c}_2\left({v}_i\right),\dots, {c}_h\left({v}_i\right)\right\} $$

Among them, *h* is the largest degree on the graph, and *h* = max_*j* = 1, …, *n*_{*d*_*j*_}.

To reduce the time complexity of calculating vector *S* (*v*_*i*_) elements, the recursive eq. (10) can be used:

10 $$ {s}_k\left({v}_i\right)=\left\{\begin{array}{l}\sum \limits_{v_j\in N\left({v}_i\right)}w\left({v}_i,{v}_j\right)\kern16.5em if\kern1em k=1\\ {}{s}_{k-1}\left({v}_i\right)-\sum \limits_{v_j\in {N}_{k-1}\left({v}_i\right)}w\left({v}_i,{v}_j\right)\kern7.25em if\kern1.5em k>1\end{array}\right. $$

Here, *S*_*k*_ (*v*_*i*_) is the *k*-th index value of vector *S* (*v*_*i*_), and *N*_*k*_ (*v*_*i*_) is the set of neighbor vertices whose degree is *k*.

Given the cumulative function vector of node *v*_*i*_, its cumulative centrality is expressed as eq. (11):

11 $$ CMC\left({v}_i\right)=\sum \limits_{k=1}^h{p}^{1+k\ast \frac{p}{r}}\ast {s}_k\left({v}_i\right) $$

In this formula, *p* and *r* are two adjustable parameters, and the value of *p* is between 0 and 1. Because there is a larger cumulative value in the lower degree than in the higher degree and in the higher-order cumulative value of many nodes, equation (11) uses the parameter ${\mathrm{p}}^{1+\mathrm{k}\ast \frac{\mathrm{p}}{\mathrm{r}}}$ to multiply the lower-order cumulative value by a larger number. This ensures that the lower-order cumulative value is more effective and has a stronger expansion and recognition ability in the regulation of node centrality.

(3) The extended *h*-index centrality *EHC*(*v*) of a node is determined according to the cumulative centrality of its neighborhood.

Formula (12) can be used to determine the extended *h*-index centrality *EHC*(*v*) of nodes by iteration:

12 $$ {\displaystyle \begin{array}{l}{EHC}^{(0)}(v)= CMC(v)\\ {}\\ {}{EHC}^{\left(t+1\right)}(v)=\sum \limits_{u\in N(v)}w\left(v,u\right).{EHC}^{(t)}(v)\end{array}} $$

(4) Calculate the centrality of all nodes and arrange them in order. *N* nodes with larger centralities are the essential nodes.

**Fig. 4:**
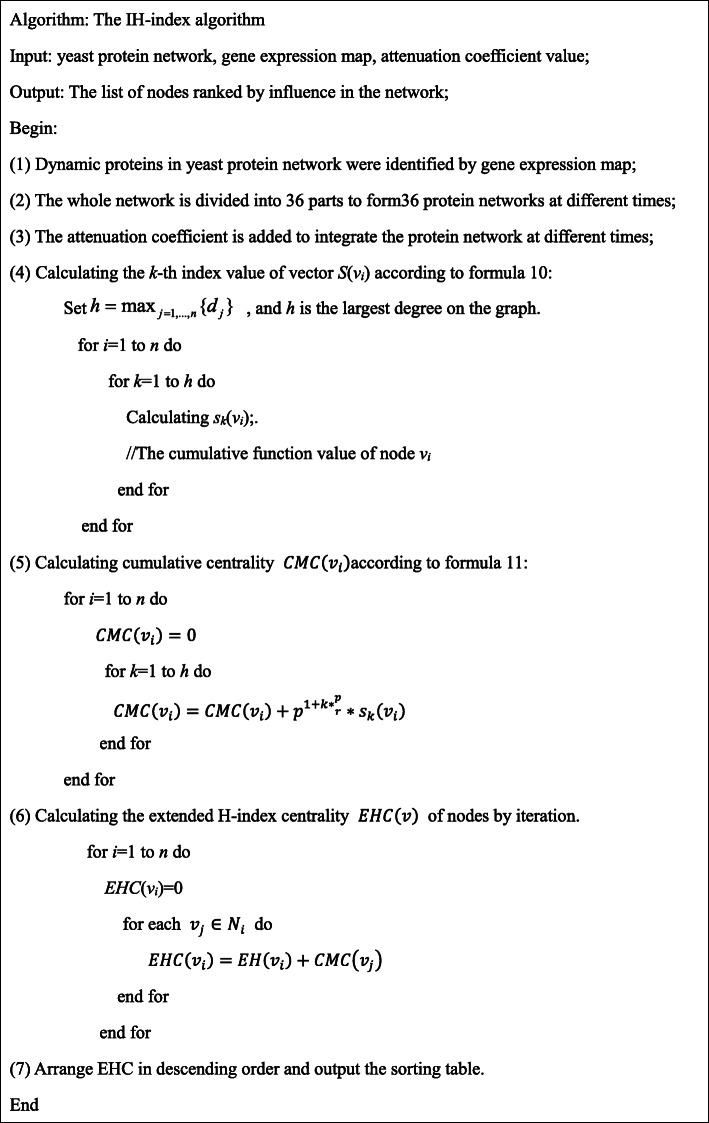
The IH-index algorithm process

The process of our proposed IH-index algorithm is shown in Fig. 4.

## Results

### Experimental data

**Fig. 5:**
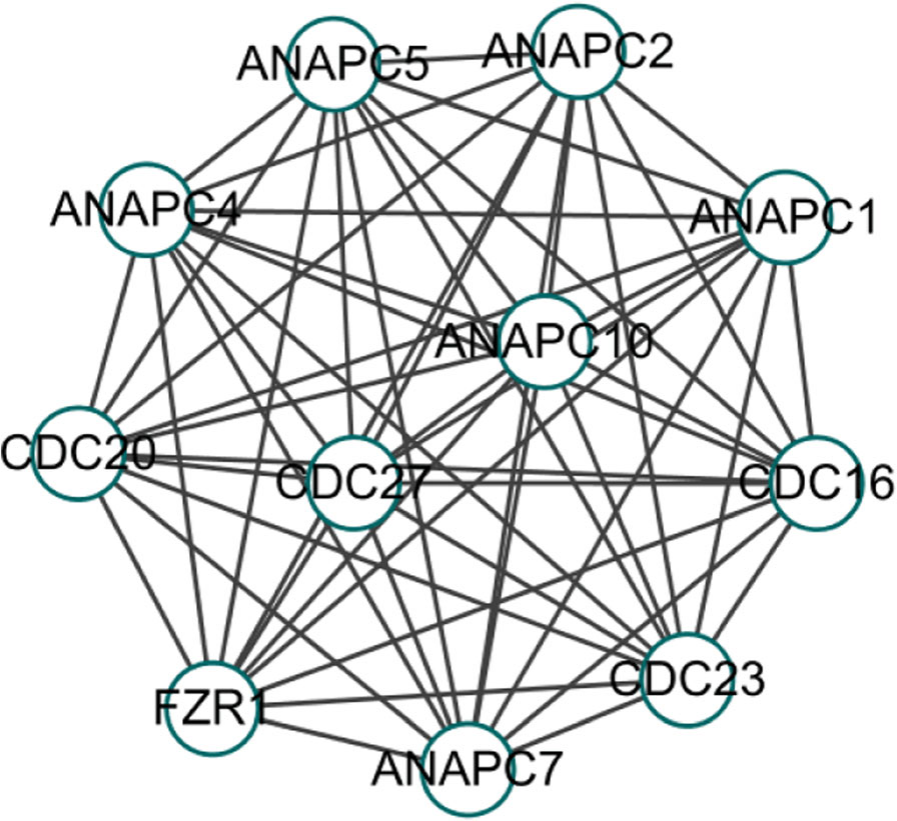
Part of the yeast protein network

The following data were used in the experiment:

1. Gene expression data GSE3431 [19]; the corresponding matrix contained 6470 lines, and each line represented the corresponding expression data of a different gene.
2. Yeast protein network in DIP [20], which includes 5093 proteins and 24,743 edges. We processed the network and extracted a portion of the nodes in the evolution process, as shown in the composition diagram in Fig. 5.
3. 1285 essential proteins obtained from the datasets MIPS [21], SGD [22], DEG [23] and SGDP [24].

### Experimental results

#### Parameter selection experiments

First, the attenuation coefficient was tested. The dynamic protein network was divided into 36 moments, and the attenuation coefficients were compared with different values. The proposed algorithm is abbreviated as the IH-index.

The SIR extension model [25] was used to evaluate the accuracy of this method in determining the node expansion capability and sorting the nodes. For this reason, the diffusion process was simulated by SIR, and the real ranking table σ was generated. In the SIR process, each node can be in one of three states: susceptibility (S), infection (I) or recovery (Re). After applying necessary changes to the node states, the node state Re was considered as the extension capability of node *v*_*i*_. The scalability of each node was calculated through repeated processing, and the ranking table σ was obtained.

After calculating the values in table σ, the sorting Table *R* can be generated by using various methods. The higher the correlation between the two ranking tables, the higher the accuracy of the corresponding methods in specifying the node expansion capability. For this reason, the Kendall correlation coefficient τ (0 ≤ τ ≤ 1) is adopted:

13 $$ \tau \left(\sigma, R\right)=\frac{n_c-{n}_d}{n\left(n-1\right)/2} $$

where *n*_*c*_ and *n*_*d*_ denote the number of consistent and inconsistent pairs of nodes in the two sorting tables, respectively, and *n* denotes the size of the sorting vector. The larger the Kendall correlation coefficient τ value, the closer the relationship between the two tables σ and *R*, and the more accurate the proposed algorithm for calculating the essential degree of the dynamic proteins.

**Fig. 6:**
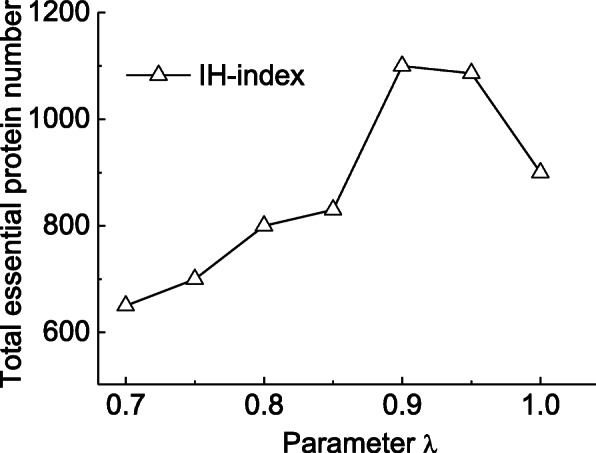
The effect of the attenuation coefficient on the number of essential proteins

**Fig. 7:**
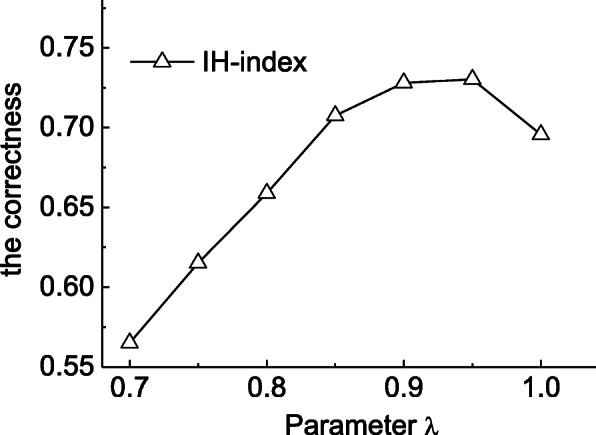
The change in correctness as the attenuation coefficient changes

Figures 6 and 7 show that the number of identified essential proteins and the accuracy of the identification change when the attenuation coefficient is altered. By synthesizing the two experimental results, we found that the attenuation coefficient ranged from 0.9 to 0.95, and the number of identified essential proteins found was optimized. Therefore, the attenuation coefficient was set to 0.92.

Next, the effect of parameters *p* and *r* on the results of the search algorithm was investigated using the yeast protein dataset by applying the Kendall coefficient.

**Fig. 8:**
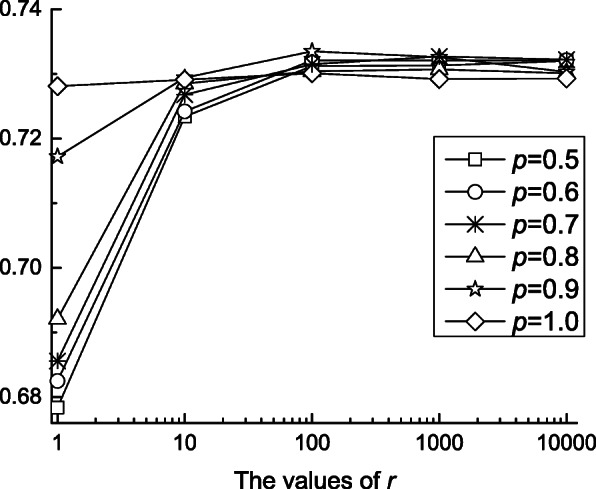
Kendall coefficient values corresponding to changes in *p* and *r*

Figure 8 shows that the value of the Kendall correlation coefficient changed slightly with the change of parameters *p* and *r*. The maximum value of τ was obtained when *p* = 0.9 and *r* = 100. Thus, the following experiments were carried out for the case of *p* = 0.9 and *r* = 100.

#### Experimental results of dynamic protein network models based on attenuation coefficients for different algorithms

To verify the performance of dynamic protein networks based on the attenuation coefficient, different algorithms were used to identify essential proteins in the established networks, and the results were compared.

The four essential node search methods were: Cnc+, [23] *h*-index, [26] IGC, [27] TEO, [11] RWEP [10] and IH-index, and they were run on the constructed attenuation coefficient-based protein network.

1. Monotonicity values

**Fig. 9:**
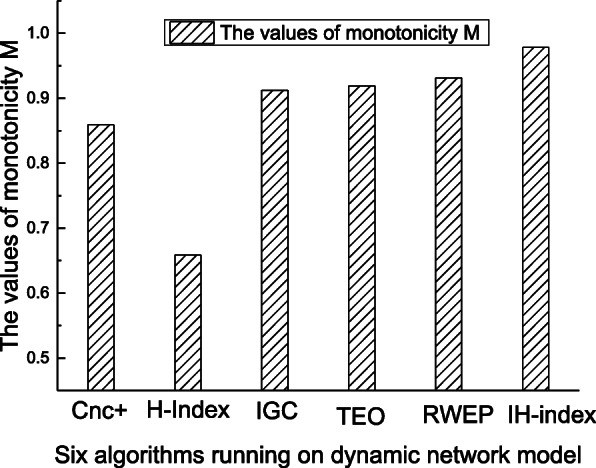
Monotonicity values of different essential protein search algorithms

We verified the monotony of the different algorithms based on the dynamic protein network to identify essential proteins. The results are shown in Fig. 9.

Figure 9 shows that the monotonicity value of the IH-index algorithm was higher than that of the other algorithms. The value was close to 1, which indicates that this algorithm has a stronger ability to recognize essential proteins.

(2) Correctness

**Fig. 10:**
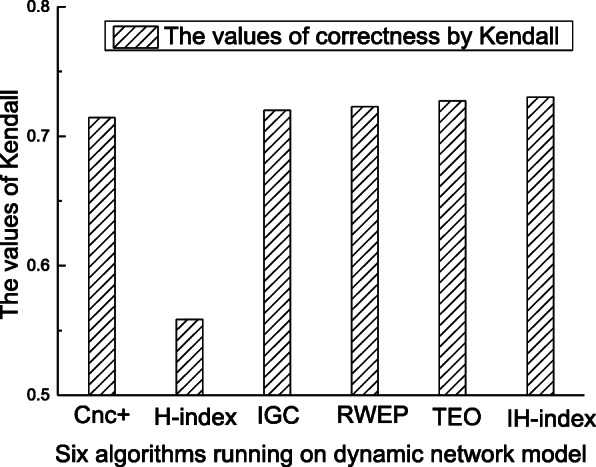
Correctness calculated by the Kendall coefficient of different essential protein search algorithms

Figure 10 shows the Kendall coefficients of two sorting tables corresponding to different algorithms. The accuracy of the IH-index algorithm in finding essential proteins was slightly higher than that of Cnc+, IGC, TEO and RWEP, and was significantly higher than that of the *h*-index.

Combined with the above two experimental results, the accuracy of the improved algorithm was also verified because the number of essential proteins found by the algorithm was the largest.

(3) The values of SN, SP, PPV, and NPV of six algorithms

To further verify the performance of the algorithm, we compared the sensitivity (SN), specificity (SP), positive predictive value (PPV), and negative predictive value (NPV) of the six different algorithms.

Table 1 presents the values of SN, SP, PPV, and NPV of the six algorithms.

**Table 1:** The values of SN, SP, PPV, and NPV of six algorithms

algorithms	Cnc+	H-index	IGC	TEO	RWEP	IH-index
SN	0.487	0.429	0.494	0.529	0.538	**0.542**
SP	0.803	0.794	0.810	0.827	0.836	**0.846**
PPV	0.456	0.376	0.472	0.481	0.493	**0.516**
NPV	0.821	0.817	0.839	0.848	0.859	**0.865**

## Discussion

Protein is an important component of all cells and tissues in the human body. The cell itself undergoes dynamic evolution in the body, such as growth, proliferation, differentiation, aging, and apoptosis. Therefore, when searching for proteins that are essential to the process of protein evolution, considering the changes in proteins at different times is consistent with the development of actual life activities. Few algorithms have considered this.

Because the algorithm experiments proposed by the predecessors are based on the protein which has been confirmed to be correct, there is no cross validation [28–30] in this paper. Next, we consider using some methods to do relevant tests. And some proteins like CDC53 appear more frequently in the whole process of biological evolution, but it is not classified as essential protein in the dataset used. Next, we will compare whether the dataset itself is overlooked, but it’s really an essential protein.

Future research should also consider that an edge plays a very small role in the network because the weight of an edge decreases with time and reaches a minimum threshold. It can be directly subtracted to save time and space. In this way, a minimum threshold is set for the weight of edges. Future research could apply this algorithm to the study of dynamic protein sequence data.

## Conclusions

To consider the influence of historical data on current protein evolution data, a dynamic protein network model based on the attenuation coefficient is proposed. In this model, rather than simply generalizing the presence or absence of proteins at each time point, a dynamic protein network modeling method based on the attenuation coefficient is used to record the changes of proteins in the process of biological evolution according to their corresponding occurrences. In the proposed model, the traditional key node search method, the *h*-index algorithm, which neglects neighbor attributes, is improved. The cumulative function is used to account for the varying degrees of the attributes of neighboring nodes, which improves the accuracy of the search for essential proteins. To verify the validity of the method, different key node search methods were applied to a dynamic protein network. The experimental results show that the model established by the IH-index method is more convenient for accurately identifying essential proteins.

## Data Availability

The datasets used during the current study are available in: DIP:http://dip.deo-mbi.ucla.edu/dip/Stat.cgi, MIPS:http://mips.helmholtz-muenchen.de/proj/ppi.
